# Asymptomatic Maternal Diseases Presenting with Symptomatic Neonatal Manifestations: A Short Case Series

**DOI:** 10.3390/children11101214

**Published:** 2024-10-03

**Authors:** Adriana Mihaela Dan, Diana Iulia Vasilescu, Sorin Liviu Vasilescu, Vlad Dima, Monica Mihaela Cîrstoiu

**Affiliations:** 1 Department of Obstetrics, Gynecology and Neonatology, Carol Davila University of Medicine and Pharmacy, 020956 Bucharest, Romania; 2 Department of Neonatology, Emergency University Hospital Bucharest, 050098 Bucharest, Romania; 3 Doctoral School, “Carol Davila” University of Medicine and Pharmacy, 050474 Bucharest, Romania; 4 Department of Obstetrics and Gynecology, Emergency University Hospital Bucharest, 050098 Bucharest, Romania; 5 Filantropia Clinical Hospital, Neonatology Department, 011171 Bucharest, Romania

**Keywords:** postpartum diagnosis, maternal–infant disease, asymptomatic diseases in pregnancy, neonatal effect of maternal antibodies, genetic diseases during pregnancy

## Abstract

It is documented that maternal diseases or treatments influence a newborn’s clinical status at birth. If a prenatal medical history is not available, or if signs or symptoms of a mother’s disease are revealed for the first time during pregnancy or postpartum, their effects on the newborn may be misattributed. Objective: The objective of this study is to emphasize the paramount importance of prenatal care, for both mothers and newborns, as a lack of maternal signs and symptoms during pregnancy does not exclude a potential severe neonatal condition. Materials and methods: We present a series of three cases of pregnant women who gave birth to very sick preterm newborns that required admission to the Neonatal Intensive Care Unit (NICU). The mothers were asymptomatic during pregnancy and unaware of their subclinical disease. The newborns’ complications, considered initially as consequences of prematurity or infection, subsequently revealed transient autoimmune disease in two of the cases (myasthenia gravis and hyperthyroidism) and a severe form of thrombophilia in the third case. Results: The newborns’ diagnosis preceded maternal diagnosis and contributed to the identification of the maternal pathology; adequate treatment was prescribed, with favorable short- and long-term outcomes. Conclusions: Prenatal exams and investigations throughout pregnancy are a good opportunity to detect subclinical diseases or predispositions. As newborns usually develop non-specific signs, one should have experience and pay attention to differentiating among etiologies. Our paper takes a reversed approach to the usual medical diagnosis pathway: from infant to mother instead of from mother to infant, proving that inter-specialty collaboration can work bi-directionally.

## 1. Introduction

Prenatal information comprising the maternal medical history, family history, pregnancy events, and paraclinical investigations is of paramount importance for neonatologists. Neonatal clinical exams are known to offer poor or non-specific signs; respiratory distress, for example, is a common manifestation for both pulmonary and non-pulmonary conditions. Severe septic shock is often signaled initially only by mottling of the skin. Extremely premature babies develop complications generated by the immaturity of their organs and systems, and it is difficult to distinguish between these and other associated co-morbidities. Worldwide medical guidelines for neonatology promote caution with interventions in extremely premature newborns, both for diagnosis and for treatment, as these may be aggressive and stressful. The neurodevelopmental care concept describes early care interventions’ influence on premature babies’ development. It has been demonstrated that from 24 to 40 weeks of gestation, the brain experiences a critical period of growth [1]. Studies have shown that the exogenous and endogenous experiences of preterm infants in the Neonatal Intensive Care Unit (NICU) can lead to developmental disorders [2,3] because patients face excessive sensory stimulation, rapid and inappropriate handling, recurrent aggressive and painful procedures, inappropriate sleep patterns, and separation from parents; all these impair the development of the immature brain [4].

Thus, for extremely low-birth-weight babies, it is important to simply support their vital functions and provide a safe environment until laboratory tests and paraclinical investigations reveal a certain etiology. Overtreatment of these babies may happen in the first days of life due to the lack of strict medical protocols. On the other hand, access to advanced investigations, such as genetic, immunologic, or molecular tests, is limited; therefore, diagnoses of rare diseases may be missed or delayed [5].

A significant help to neonatologists’ medical judgement is given by informational input from the obstetricians, as there are strong evidenced-based correlations between maternal illnesses and fetal/neonatal outcomes. The maternal medical history is crucial in neonatal outcomes. For example, Group B Streptococcus (GBS) is a common commensal in the human gut and the lower genital tract of women that is completely harmless in adults, but its presence during pregnancy can lead to important consequences for the offspring: sepsis and long-term sequelae such as meningitis have been reported [6]. Unfortunately, there are diseases affecting young child-bearing women that can be misdiagnosed and can exert potential effects on the fetus or newborn, for example, genetic or autoimmune diseases such as thrombophilia, myasthenia gravis, and Basedow–Graves’ disease.

Thrombophilia is defined as a predisposition to thrombosis, mostly due to genetic anomalies, it but can also be acquired postnatally. Pregnancy is one of the conditions that induces a hypercoagulable state by itself (decreased anticoagulant factor level); in association with hereditary thrombophilia, pregnancy represents a higher risk for localized thrombosis and venous or arterial thromboembolism. Screening for thrombophilia before or during pregnancy is not a standard of care but is recommended for women with a personal or family history of thromboembolism, during an eventful pregnancy, or after repeated miscarriages. The incidence of venous thromboembolism (VTE) in pregnancy increases by up to five times, with a rate of 0.76–1.72 out of 1000 pregnancies, but most thrombotic events occur in the puerperal period [7,8]. Inherited thrombophilia is assumed to cause up to 50% of venous thrombotic events that occur during gestation and puerperium and may have severe consequences for both the mother and the fetus [8,9,10].

Even if the maternal genetic anomaly is inherited by the fetus, thromboembolic disease is rare in childhood [11,12]. The annual incidence in children is estimated at 0.7 cases of venous thrombosis, 1.0 cases of stroke, and 0.1 cases of myocardial infarction per 100,000 population. For critically ill neonates, the prevalence of VTE is much higher (about 10% of VTEs occur in the first four weeks of life). Other conditions that increase the general risk are lower concentrations of natural inhibitors and impaired fibrinolytic activity, elevated levels of von Willebrand factor [13,14], the high viscosity of newborns’ blood due to their high hematocrit and small vascular diameter, dehydration, or a hypercoagulable state due to infection or prematurity. The use of venous or arterial central catheters is a widespread practice in NICUs and represents a risk factor for thrombosis [15].

Although encountered more frequently in newborns compared to older children, ischemic stroke is a rare diagnosis in the neonatal period in the absence of general imagistic screening. Thromboembolic events are considered to be underdiagnosed, being discovered later in life as motor impairments. Still, there is no consensus regarding the administration of preventive low doses of aspirin or anticoagulants such as low-molecular-weight heparin to pregnant women with thrombophilia. Additionally, coagulation and molecular tests are expensive and not easily available; therefore, they are not routinely recommended for all pregnant women or newborns from thrombophilic mothers [16].

In women of childbearing age, there is also a high prevalence of systemic autoimmune disorders, with possible consequences on the fetus and newborn infant due either to the disease itself or to corticoid or immunosuppressive treatment [17]. The gestational complications of autoimmune disorders are miscarriage, stillbirth, pre-eclampsia, blood clots, preterm delivery, and intrauterine growth retardation; if the maternal disease is characterized by the presence of IgG isotype auto-antibodies, these can cross the placenta, causing possible antibody-mediated damage to the fetus. In neonatal lupus erythematosus, neonatal congenital heart block is encountered, and neurodevelopmental dysfunctions (learning disabilities or attention deficit) are also described in children born to mothers with systemic lupus erythematosus [18,19]. Maternal glucocorticoid (GC) treatment does not affect the incidence or level of autoantibodies and infant symptoms, but the probabilities of preterm delivery and LBW infants are higher and more remarkable in gravidas treated with GCs [20,21].

Other neonatal autoimmune diseases involving interactions between maternal antibodies and fetal/neonatal antigens include neonatal anti-phospholipid syndrome, Behcet’s disease, neonatal autoimmune thyroid disease, neonatal polymyositis and dermatomyositis, neonatal scleroderma and neonatal type I diabetes mellitus, neonatal myasthenia gravis, and Kawasaki disease. While autoantibodies have been detected in patients with neonatal autoimmune disease, other mechanisms may play a role in the development of neonatal autoimmunity, such as fetal/maternal microchimerism and the aberrant apoptosis of fetal cells. In some of these conditions, the target antigen may remain unknown [22].

The prevalence of autoimmune diseases among pregnant women is difficult to estimate, and the neonatal clinical picture has different degrees of expression. The discrepancy between maternal and neonatal severity can be partially explained by the protective effect of α-fetoprotein, which inhibits the binding of specific antibodies to its receptors [23].

Usually, the diagnosis of maternal disease precedes the diagnosis of fetal or neonatal complications. It is not necessary to test for the presence or level of neonatal antibodies if the mother has a clear previous diagnosis and the child’s signs are suggestive of the mother’s disease. If the mother is not diagnosed, it is much more difficult to rely only on the baby’s clinical exam. However, there are some evidence-based associations, like complete atrioventricular block, which strongly suggests lupus erythematosus. Differential diagnosis, especially if the patient is born prematurely and has alterations in vital functions attributed to immaturity or an overlapping infectious disease, can be difficult, and specific treatment may be delayed.

Therefore, this study aims to underline the importance of diagnosing pathologies that are pre-existent or related to pregnancy, to anticipate and adequately treat any potential complications in the newborn.

## 2. Materials and Methods

We present three cases of pregnant women without any diagnosed pathology associated with pregnancy who gave birth to 4 premature newborns (one twin pregnancy) that developed severe symptoms postnatally: seizures (#1), respiratory distress and hypotonia (#2), and arrhythmia (#3,4), all of which required admittance to the NICU. The onset of the symptoms was immediate after birth for baby #2 and after a certain period in the other cases, raising problems in terms of differential diagnosis and management. All cases were disproportionately affected considering their prenatal history or gestational age and required specific treatment to control their symptoms. The baseline characteristics of the newborns and mothers are summarized in Table 1.

**Figure 1:**
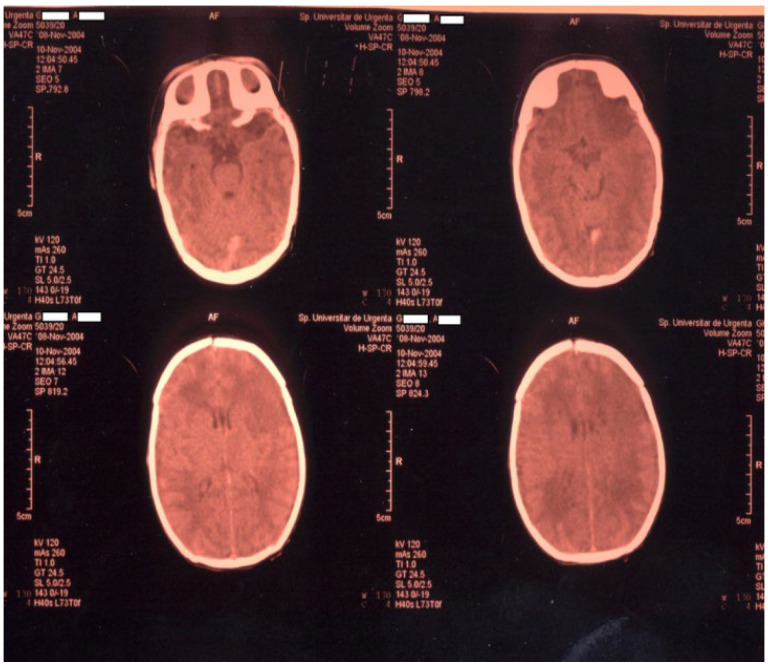
CT examination for newborn #1.

**Figure 2:**
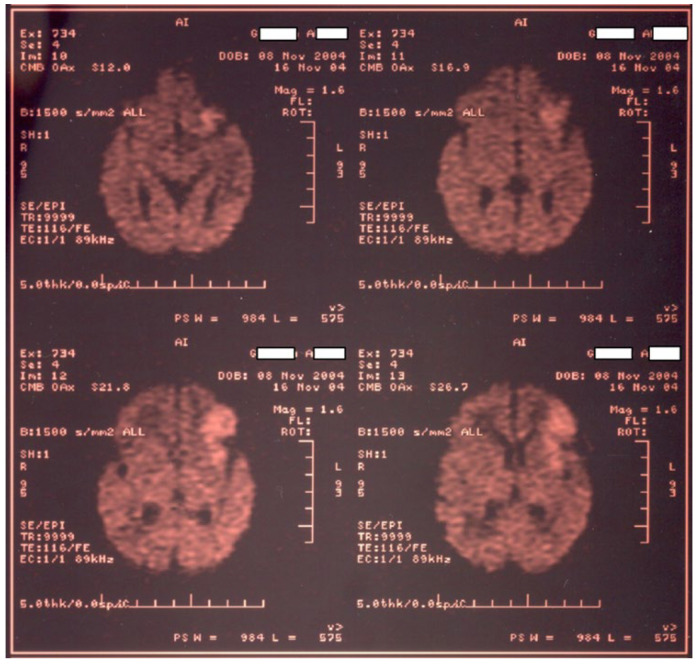
MRI examination for newborn #1 confirms a large right frontal–parietal infarction.

Newborn #1 (3000 g/50 cm, female) was born at 36–37 weeks of gestation by caesarean section after an uneventful pregnancy and was the first child of a healthy mother. The Apgar score was 9, the transition to the extrauterine environment was smooth, and the clinical examination after birth was normal. At 36 h of life, the baby developed tonic–clonic seizures of the left hemibody associated with cyanosis, apnea, and bradycardia and was admitted to the NICU. The baby was tested for metabolic causes of the seizures; a lumbar puncture and inflammatory panel and bacteriologic tests were performed, and phenobarbital was started. Because of recurrent apneic spells, respiratory support was initiated, and imagistic investigations were ordered. Head ultrasonography raised suspicion of a large right frontal–parietal infarction, confirmed later through Computer Tomography (CT) and Magnetic Resonance Imaging (MRI)—see Figure 1 and Figure 2. Echocardiography revealed no abnormalities. Laboratory tests were all within normal limits, except a mild thrombocytopenia (105,000/mmc).

Within the differential diagnosis, we considered maternal chorioamnionitis, hypoxic ischemic encephalopathy, vascular malformations, cardiac lesions, trauma, and a hereditary coagulopathy. We performed molecular tests for both mother and infant that revealed a heterozygote G1691A mutation on the gene responsible for synthesizing factor V Leiden. A less frequent condition related to ischemic stroke in children and adults, though not documented in newborns, is sickle cell disease; this was also excluded [24,25].

Despite the higher incidence of thrombosis in the neonatal period compared to later childhood, most of the guidelines for neonatal thrombosis do not recommend systematic thrombolysis or anticoagulants [26]; therefore, our therapeutic approach was conservative.

The newborn was weaned from the ventilator after 72 h and continued with phenobarbital (maintaining the same dose) for up to 3 months, according to the neurologist’s recommendation. No thrombolytic agent or heparin was given, but long-term physical therapy for motor deficits and spasticity of the left hemibody was required. At 5 years of age, the child’s outcome has been favorable, with recovery ad integrum; remarkably, the child is left-handed.

The mother had a second pregnancy after two years and delivered a second female baby at term (3500 g/50 cm, Apgar score of 10) with no pathology. Genetic tests in this case showed the same genetic mutation found in her sibling. The prenatal diagnosis of thrombophilia and the fact that low-weight heparin was administered during the pregnancy may have made the difference.

Newborn #2 provided a challenging situation: a premature female baby (30 weeks of gestation) with intrauterine growth restriction (1600 g/43 cm) born to an uninvestigated mother with a negative familial or personal medical history, with oligoamnios and opalescent amniotic fluid. The baby had Apgar scores of 2 at 1 min and 5 at 5 min (general tone and respiratory effort were significantly decreased), requiring resuscitation (positive pressure ventilation and oro-tracheal intubation) for apnea resistant to continuous positive airway pressure (CPAP) and the administration of caffeine. After admission to the NICU, the baby was mechanically ventilated with low FiO2 and low pressure. A surfactant was not administered since the clinical and imagistic data were not consistent with respiratory distress syndrome. The clinical findings included hypotonia, hyporeactivity, a lack of archaic reflexes, and signs of apparent dehydration (low skin turgor, depressed fontanelle), feeding intolerance, and a discreet facial dysmorphism. Laboratory tests indicated positive inflammatory syndrome, mild hyponatremia (130 mEq/mL), and positive bacteriological results (*Escherichia coli*). A head ultrasound identified a grade II intraventricular hemorrhage, a chest X-ray excluded a pulmonary condition, and echocardiography showed persistent ductus arteriosus (PDA) without hemodynamic significance. The EKG was normal, and the aEEG recorded normal cerebral activity. Differential diagnoses included asphyxia, respiratory depression due to prematurity, intracranial hemorrhage, early neonatal sepsis, suprarenal insufficiency, and genetic syndrome (Prader–Willi was suspected). Another considered cause for the hypotonia was myasthenia gravis, but a clinical examination of the mother was perfectly normal, and none of the usual investigations performed pre- and post-caesarean section revealed any abnormalities; she was discharged home at 4 days after birth. The newborn was treated with broad-spectrum antibiotics, caffeine, prolonged total parenteral nutrition (TPN) with correction of biochemical deficits, fresh frozen plasma (for intracranial hemorrhage), hydrocortisone hemisuccinate (for the clinical suspicion of SR insufficiency), and intravenous nonspecific human immunoglobulin (i.v. Ig) as an adjuvant therapy for sepsis. After more than two weeks of intensive treatment, inflammatory tests and cultures were negative, biochemical tests were normalized, lumbar puncture results were negative, and head US results were stable, but the clinical picture was not improving, except for a slight alleviation after the cure with I.V. human immunoglobulin (3 × 0.5 g/kg/day).

At that time, the mother had been re-admitted to the hospital with severe bronchopneumonia and respiratory failure (she was mechanically ventilated) and neurological alterations (dysphonia, dysphagia, difficulties in swallowing and speaking, tetraparesis). She tested positive for SARS-CoV-2 infection. Her condition was initially considered to be caused by the coronavirus, and all therapeutic efforts were conducted in this direction. She received Remdesivir, broad-spectrum antibiotics, corticotherapy, and plasmapheresis for cytokine removal (a therapeutic approach used in the second phase of coronavirus infection at that time). After the second session of plasmapheresis, the patient recovered from a respiratory point of view, but she continued to experience major fatigue and motor deficits with circadian variations that raised suspicion of myasthenia gravis. Miostin was added to the treatment and rapidly alleviated the patient’s condition. Serologic tests were positive for anti-acetylcholine receptor antibodies after the disease had been controlled. The baby showed slow progressive improvement in her clinical state before any specific treatment was used, probably due to a natural decrease in the level of antibodies (she was already almost 4 weeks of age). Spontaneous respiratory effort allowed for extubation at one month of age, feeding was improved by efficient sucking and swallowing reflex and tone, and reactivity significantly increased. Long-term follow-up of the case showed normal growth and development without chronic treatment, and despite the long-term mechanical ventilation, associated sepsis, and complications, the child presents no neurologic dysfunctions. Although rare, there are documented unfavorable outcomes for babies born to mothers with myasthenia gravis, like arthrogryposis multiplex congenita (AMC) or fetal acetylcholine receptor antibody-associated disorders (FARADs), or even neonatal demise [27,28].

Newborns #3 and #4 were home-delivered premature female twins (28 weeks of gestation, 900 g and 890 g) referred to our hospital from another clinic and were the result of an uninvestigated pregnancy. They developed respiratory distress syndrome requiring mechanical ventilation and surfactant administration, with difficult recovery due to associated early neonatal sepsis with Corynebacterium pseudotuberculosis; despite treatment with antibiotics, oral feeding, and parenteral nutrition, our patients had poor growth and intermittent fever spells associated with tachycardia. Initially, these signs were attributed to infection, but their persistence beyond the first week of life and treatment, in combination with negative bacteriological tests, led to a suspicion of neonatal hyperthyroidism, although the mother did not have any documented related condition. The values for TSH were extremely low, and the thyroxine and tri-iodothyronine levels were above normal limits. No somatic clinical sign of hyperthyroidism was present (such as exophthalmia or goiter). The patients were treated with Tyrosol and Propranolol in doses adjusted over time for their age and weight and followed up by the endocrinologist; after 7 days of treatment, the clinical symptoms disappeared and the babies began to gain weight, while hormonal tests came back normal (the treatment was discontinued at 3 months of age).

During the children’s hospitalization (day 5 after birth), their mother presented with symptomatic tachyarrhythmia (dyspnea, oedema, and chest pain) and was admitted to the cardiology department of our hospital with a diagnosis of cardiac failure class IV NYHA (ejection fraction 35%) and intracardiac thrombosis with possible cardioembolism, later attributed to thyrotoxicosis. Her TSH level was 0.001 UI/mL, and she was given Tyrosol and Lugol solution in decreased doses along with cardiologic treatment (anticoagulants, BB, IEC, and diuretics). This correlation between maternal and fetal hyperthyroidism raised a suspicion of Basedow–Graves’ disease, which was later confirmed by the results of serological testing. Both the mother and her babies had a favorable short-term outcome but were lost to follow-up after 3 months.

## 3. Discussion

We chose to present these three different complex and surprising cases because all of them had an unusual but common sequence: with no prenatal information or a negative history, prematurity-related complications, and poor responses to standard care, the babies were diagnosed before their mothers, and information from the neonatologists served to diagnose the mothers. Because the three medical conditions have different pathogeneses and clinical pictures, we will discuss them separately to emphasize their particularities.

Newborn #1 was quite a rare case of an ischemic event due to maternal thrombophilia; the medical literature reports a low incidence of fetal or neonatal events, the most cited association being between miscarriages and genetic coagulopathies [8].

Most perinatal strokes are ischemic and affect the left cerebral hemisphere, in the territory of the middle cerebral artery [26]. In our case, the infarct was in the frontal–parietal right cortical region. We could not identify any of the supplementary risk factors for thrombosis in this case.

In 2015, we performed a retrospective study (unpublished) covering a 5-year period (2010–2014) regarding the postnatal effects of maternal thrombophilia observed in our clinic; we analyzed all medical records of our patients (mothers and children) to discover associations between maternal genetic anomalies and neonatal outcomes. The results showed no statistically significant correlation between maternal thrombophilia and specific neonatal pathologies in treated cases. From 19.910 births that took place in the University Hospital in the study period, we identified 2548 pregnant women with genetic thrombophilia (12.8%). The number of preterm births was not statistically significantly higher among mothers with thrombophilia, although more babies were born before 37 weeks of gestation in the group of women with thrombophilia. The weight at gestation was similar for babies born to mothers with and without thrombophilia. The prevalence of neonatal complications was not higher in the group of children from mothers with thrombophilia. None of the cases from that period were diagnosed with thrombotic complications (neither the mothers nor the babies). The women included in the study were either not treated at all (if they were asymptomatic and had a negative family history) or received aspirin or low-molecular-weight heparin (if they had been diagnosed with intrauterine growth restriction (IUGR) or other signs of fetal distress). There was no statistical difference regarding neonatal outcomes between treated and not-treated mothers, leading to the conclusion that routine prophylactic treatment during pregnancy is not compulsory. In this context, we consider the presented case very interesting and unexpected: ischemic stroke in a term baby without any associated maternal pathology during pregnancy or the perinatal period is a rare condition. In some cases, events like this may be asymptomatic, discovered by a routine imaging investigation, or can be expressed later in life when motor development progresses asymmetrically. The prognosis in most of these cases is poor, with motor deficits depending on the size, localization, and time of diagnosis and initiation of kinetotherapy. Despite the severe clinical picture, our case had a favorable outcome due to the rapid interventions. Moreover, during a second pregnancy in the same woman, preventive anticoagulation was administered, and despite inheriting the same genetic mutation, the sibling had no anomalies in her coagulation status.

In case #2, the most important clinical sign was severe neurologic depression that was disproportionate with the grade of prematurity or intraventricular hemorrhage; sepsis or meningitis could also have been responsible for the symptomatology, but a lumbar puncture was negative, and antibiotics did not influence the clinical course in that matter. Associated hyponatremia, signs of dehydration, and slightly dysmorphic features led us to consider metabolic or genetic rare diseases, which were then excluded by the normal results of specific tests. Myasthenia gravis was initially excluded from the differentials as there was no positive maternal or family history, and congenital myasthenia gravis (a neonatal genetic condition—autosomal recessive inherited) is an extremely rare and lifelong condition. The use of I.V. immunoglobulin in our case as an adjuvant therapy for sepsis and/or meningitis might have been beneficial for the baby, as it could bind some of the circulating specific antibodies [29]. Prolonged cardiorespiratory support and parenteral nutrition were essential to ensure the baby’s well-being until diagnosis and the natural clearance of the antibodies. There is also juvenile myasthenia gravis, which resembles the adult form but is not expressed in the neonatal period [29]. Even when the mother is diagnosed with myasthenia gravis, only 10–20% of infants develop postnatal symptoms [30].

An interesting fact is that the mother’s disease only became clinically patent postpartum, in the context of a respiratory infection with SARS-CoV-2. Although not consistently, studies have demonstrated that pregnancy or the peripartum period can exacerbate myasthenia gravis symptoms [27,31,32]. Serological testing in our case was positive for anti-acetylcholine antibodies (55.5 nmoli/L compared to the normal limit of <0.25 nmol/L) and negative for anti-MUSK antibodies. The level of antibodies (more than 20 times the normal) may have influenced the severity of the disease. This case is peculiar because the onset of neonatal symptomatology was immediate after birth (usually, symptoms appear after 2–3 days of life) and the disease had such severe clinical expression in the newborn, affecting respiratory muscles, while the mother was asymptomatic. Facial dysmorphism, which has been interpreted as syndromic, is a sign of diplegia [29].

Our previous recent experience with transient myasthenia gravis consists of self-limited asymptomatic or mildly symptomatic cases, with no vital risk. The most severe clinical expression among our patients was represented by difficulties in sucking and swallowing, requiring short-term specific treatment (Miostin). Mothers were diagnosed antepartum, with or without chronic treatment. All of this demonstrates the great variability of the clinical course and picture of this disease. Due to the physiological effects of acetylcholine in the human body and the presence of the receptors in many anatomic structures of the body, the symptomatology can be mild or life-threatening, but it is usually self-limited within weeks or months, as in the presented case [33].

Newborns #3 and #4 were premature female twins (28 weeks of gestation) born to a mother with no prenatal care and with risk factors for early-onset sepsis, with a poor response to standard postnatal care. The persistence of fever, episodes of tachycardia, and unsatisfactory weight gain despite enteral and parenteral nutrition raised a suspicion of hyperthyroidism, although a clinical exam showed no exophthalmia or goiter, and no maternal symptomatology or history seemed to support a thyroid disorder. Hormonal testing in both babies confirmed hyperthyroidism (low TSH, elevated T3 and T4), although premature babies usually have transient hypothyroidism in the first weeks of life [34]. It may be that the mother’s disease was only recently decompensated, or the decreased thyroid function due to prematurity may have counterbalanced the effect of maternal stimulating antibodies on the newborns, and the symptomatology was not obvious from the beginning.

The infants were transferred to our hospital for the treatment of complications of prematurity and were not accompanied by their mother, so we could not observe her facial features; a certain degree of exophthalmia, which was noticed after she was admitted to our hospital for a severe cardiac condition, was proven to be caused by thyrotoxicosis. Such an abrupt onset and severe clinical course is unusual for Basedow–Graves’ disease. The concordance of hormonal abnormalities in both the mother and infants raised a suspicion of a maternal autoimmune disease before serological tests (specific antibodies) to confirm it.

In our experience, we had a previous case of a newborn whose mother had been diagnosed and treated for Basedow–Graves’ disease before pregnancy, having an elevated level of TRAB antibodies. The baby was born at 35 weeks of gestation, with typical signs of hyperthyroidism: exophthalmia, goiter, hypertonia agitation, myoclonus, feeding difficulties, and slow weight gain; fever and sinus tachycardia were also noted. Given this documented and treated pathology of the pregnancy, the baby was approached immediately after birth with specific investigations and treatment, with rapid and good clinical outcomes.

This case was atypical since the onset of symptoms was later than usual and had a poor clinical picture: tachycardia and failure to thrive. Infants born to a mother with active Graves’ disease, if untreated, usually present clinical manifestations in the first 1–2 days of life. In contrast, infants born to a mother who has received antithyroid treatment can be euthyroid or even hypothyroid at birth, and their clinical manifestation of neonatal Graves’ disease can appear up to two weeks after birth.

Neonatal manifestations are more likely to appear in cases with higher maternal stimulatory Trab concentrations in the third trimester, and in the absence of diagnosis and treatment, neonatal thyrotoxicosis can increase the mortality rate to 15%, especially through cardiovascular complications [35]. Our patients were diagnosed in the second week of life, but prompt intervention led to a favorable short-term outcome, despite supplemental risk factors (prematurity and sepsis) and loss to follow-up.

## 4. Conclusions

Neonatal pathologies due to maternal genetic and autoimmune diseases are rather rare conditions. There are not enough evidence-based guidelines for standard postnatal care, and, therefore, these pathologies represent a concern for the neonatologist. Their impact on the newborn is difficult to anticipate as the clinical picture in the first days of life may not be relevant, but they can still influence short- or longer-term outcomes. Fortunately, most of the autoimmune pathologies at the neonatal stage are transient and self-limited, but it is difficult to estimate the intensity and the duration of the symptoms. Genetic diseases such as thrombophilia do not require routine extended investigations or treatment but should be considered as risk factors for serious complications. When the maternal disease is not documented or there is an asymptomatic pregnancy, the management of neonatal patients becomes more difficult, and it takes longer to identify the appropriate intervention. We emphasize the importance of thorough prenatal care and a close interdisciplinary approach, as more information gathered from different sources can provide a relevant diagnostic argument that can sometimes replace expensive or unavailable medical investigations.

## Figures and Tables

**Table 1:** Characteristics of the 4 newborns and associated pathologies of their mothers.

	Newborn 1	Newborn 2	Newborn 3	Newborn 4
Gender	F	F	F	F
Gestational age, weeks	36	30	28	28
Birth weight	3000 g	1600 g	900 g	890 g
Apgar score	9/9	2/5	unknown	unknown
Delivery	cesarean section	cesarean section	home delivery	home delivery
Newborn’s first symptoms	seizures at 36 h of life	hypotonia and apnea at birth	tachycardia at 72 h of life	tachycardia at 72 h of life
Investigations during pregnancy	yes	no	no	no
Mother’s disease	thrombophilia	myasthenia gravis	Basedow–Graves’	Basedow–Graves’
Time of diagnosis of the maternal disease	5 days after delivery	2 weeks after delivery	5 days after delivery	5 days after delivery
Postpartum clinical expression of maternal disease	none	neurologic depression	cardiac failure	cardiac failure
Method of diagnosis of maternal disease	genetic tests	anti-Ach receptor antibodies	TSH, fT4, Trab antibodies	TSH, fT4, Trab antibodies
Infants’ medication at discharge	phenobarbital for 3 months	No	Tyrosol and Propranolol for 3 months	Tyrosol and Propranolol for 3 months
Socioeconomic maternal status	26 yo	21 yo	24 yo	24 yo
	GIPI	GIPI	GIPII	GIPII
	married	married	married	married
	higher education	secondary school	no education	no education
	urban	rural	rural	rural

## Data Availability

The data presented in this study cannot be found elsewhere.
